# Bayesian Cross-Validation Comparison of Amino Acid Replacement Models: Contrasting Profile Mixtures, Pairwise Exchangeabilities, and Gamma-Distributed Rates-Across-Sites

**DOI:** 10.1007/s00239-022-10076-y

**Published:** 2022-10-07

**Authors:** Thomas Bujaki, Nicolas Rodrigue

**Affiliations:** 1 Department of Biology, Institute of Biochemistry, Carleton University, Ottawa, Canada; 2 School of Mathematics and Statistics, Carleton University, 209 Nesbitt Biology Building, 1125 Colonel By Drive, Ottawa, ON K1A 0C6 Canada

**Keywords:** Phylogenetics, Pattern heterogeneity, Dirichlet process, Finite mixture, Empirical models

## Abstract

Models of amino acid replacement are central to modern phylogenetic inference, particularly so when dealing with deep evolutionary relationships. Traditionally, a single, empirically derived matrix was utilized, so as to keep the degrees-of-freedom of the inference low, and focused on topology. With the growing size of data sets, however, an amino acid-level general-time-reversible matrix has become increasingly feasible, treating amino acid exchangeabilities and frequencies as free parameters. Moreover, models based on mixtures of multiple matrices are increasingly utilized, in order to account for across-site heterogeneities in amino acid requirements of proteins. Such models exist as finite empirically-derived amino acid profile (or frequency) mixtures, free finite mixtures, as well as free Dirichlet process-based infinite mixtures. All of these approaches are typically combined with a gamma-distributed rates-across-sites model. In spite of the availability of these different aspects to modeling the amino acid replacement process, no study has systematically quantified their relative contributions to their predictive power of real data. Here, we use Bayesian cross-validation to establish a detailed comparison, while activating/deactivating each modeling aspect. For most data sets studied, we find that amino acid mixture models can outrank all single-matrix models, even when the latter include gamma-distributed rates and the former do not. We also find that free finite mixtures consistently outperform empirical finite mixtures. Finally, the Dirichlet process-based mixture model tends to outperform all other approaches.

## Introduction

Most studies aimed at determining deep phylogenetic relationships utilize large alignments of amino acid characters. Phylogenetic analyses at this level traditionally invoked an empirical model of the amino acid replacement process, from the early counting-based approaches of Dayhoff et al. (1978), Jones et al. (1992), and others, to the maximum-likelihood-based matrices, such as those of Whelan and Goldman (2001) and Le and Gascuel (2008). Biologically motivated by the idea that pairwise amino acid exchangeabilities—and to a lesser extent amino acid frequencies—could be broadly similar across many contexts, the rationale behind an empirical matrix was to construct a generalized amino acid replacement model, where the parameters were reliably inferred from a large data set, and thus avoiding the repeated fitting of 190 exchangeabilities and 20 frequencies on each subsequent data set. This prudent approach was warranted when the alignment available for a given phylogenetic problem was small. Over time, as data sets grew in size, it became a common practice, at first, to treat the 20 amino acid frequencies as free parameters, combined with empirical exchangeability parameters. Eventually, data sets reached several thousand characters, such that a full amino acid-level general-time-reversible (GTR) matrix could often be reliably inferred, and even richer models could be considered.

These developments were generally explored in a context allowing for a basic site heterogeneity of overall rates (i.e., without regard to the nature of amino acid replacements). The most common approach to this end has been to invoke site-specific rates acting as branch length multipliers; these rates are treated as random variables following a gamma distribution of mean 1, with variance governed by an additional parameter of the inference (Yang 1993, 1994). Under this gamma-distributed rates model, the likelihood function at each site takes the form of an integral of the likelihood score over all possible rates values, weighted by the prior density under the gamma law. The integral has no analytical solution, however, and is rather approximated by discretizing the gamma law into 4 or 8 equally weighted categories. As such, the likelihood function becomes an average of the likelihood score over 4 or 8 different rate values (Yang 1994), giving a model form analogous to that of a finite mixture (see, e.g., Neal 2000for an exposition on mixture models).

Other approaches to accommodating across-site heterogeneity in the amino acid replacement process have focused on utilizing different rate matrices for different classes of sites; the goal being to capture *pattern heterogeneity*, i.e., a variation across sites in the types of amino acid replacements. Earlier strategies consisted of either using a predetermined grouping of sites sharing structural features, with sites in each group assigned to a common rate matrix (e.g., Goldman et al. 1996; Liò and Goldman 1999), or using biochemically predefined rate matrices, and letting sites “choose” one among those available (e.g., Koshi and Goldstein 1998, 2001). The next natural extension of amino acid replacement models was to jointly explore mixture-like gamma-distributed rates and mixtures of replacement rate matrices.

The most important subsequent development along these lines came with the introduction of the CAT model (Lartillot and Philippe 2004). Named after its effective CATegorization of amino acid frequency profiles, the approach represents an extreme in mixture modeling flexibility and computational techniques: the amino acid profiles, their weights, and the number of profiles, are modeled as a Dirichlet process (Ferguson 1973; Antoniak 1974). Different perspectives to the Dirichlet process have been adopted for its practical implementation, using various Monte Carlo algorithms. A first example is the “Chinese restaurant” algorithm, where the number of components of the mixture is a latent variable, such that the likelihood function’s parameterization changes over the course of the Monte Carlo sampling (Lartillot and Philippe 2004). Another perspective on the Dirichlet process is the “stick-breaking” process, with a likelihood function expressed as a weighted average over an infinite number of mixture components, ultimately truncated as part of the approximation protocol (Lartillot et al. 2013). The Dirichlet process is sometimes classified as a non-parametric approach, and is often referred to as an infinite mixture model.

The CAT model has had an important impact on phylogenetic inference, in accounting for potential homoplasies, which in turn make it more resistant to long-branch-attraction artifacts (Lartillot et al. 2007). Implemented in a Bayesian framework relying on Markov chain Monte Carlo sampling, it can be computationally demanding, and is sometimes susceptible to convergence difficulties (Lartillot 2020). These issues stimulated the development of simpler mixture modeling approaches, inspired by the rationale of the classic empirical amino acid replacement matrices: construct a finite mixture of amino acid profiles, with a pre-determined number of components, and infer the profiles and their weights from a large data set (e.g., Quang et al. 2008; Schrempf et al. 2020). The resulting empirical mixture model could then be applied as-is in subsequent phylogenetic analyses, or given some flexibility by re-inferring the weights of mixture components from the data set of interest. Susko et al. (2018) proposed a composite-likelihood approach where a finite mixture could be inferred directly from a specific data set of interest. In a Bayesian framework, free finite mixtures of amino acid profiles, with parameters sampled from their posterior distribution, remain virtually unexplored.

Evaluations of the predictive power of mixture models were first mainly focused on comparisons against single-matrix approaches. Lartillot and Philippe (2004, 2006) used Bayes factors, and later, cross-validation (Lartillot et al. 2007), showing that the CAT model outperforms empirical and GTR models. Quang et al. (2008) used information criteria to compare empirical mixtures against empirical single matrices. More recently, Susko et al. (2018) compared their free finite mixture models with empirical mixtures on the basis of likelihood scores. Li et al. (2021) used cross-validation to contrast CAT against a finite mixture model invoking 60 components. However, we still lack a comprehensive study of how infinite, free finite and empirical mixture models compare to one another. The relative importance of having free exchangeability parameters in each of these contexts is largely unknown. Finally, while the gamma-distributed rates-across-sites approach is widely recognized as an important element, quantifying its contribution to the predictive power of a model in comparison with other elements, such mixtures of amino acids or free amino acid exchangeabilities, is also unexplored.

Among the model comparison methods available, only Bayesian cross-validation is currently available in a computationally tractable framework for all the models that interest us here. The approach directly measures a model’s predictive power, that is, its ability to anticipate the features of previously unseen data, having been trained another dataset. It should be noted that by “predictive power” we are not actually concerned with predicting amino acid sequences for a particular task. One general objective of comparing models is to find the best approximation of the true (unknown) data-generating process. This is also the general objective of information criteria and Bayes factors, which can also be framed as comparing predictive power: cross-validation and AIC, for instance, have an asymptotic equivalence (see Stone 1977) and Bayes factors correspond to ratios of prior predictive probabilities.

Here, we perform a detailed comparison of a wide set of amino acid replacement models based on Bayesian cross-validation. Using five previously published data sets, we contrast all aspects of the models, including free amino acid exchangeabilities, empirical finite, free finite and infinite mixtures of amino acid profiles, and gamma-distributed rates-across-sites, in all combinations.

## Results and Discussion

**Fig. 1:**
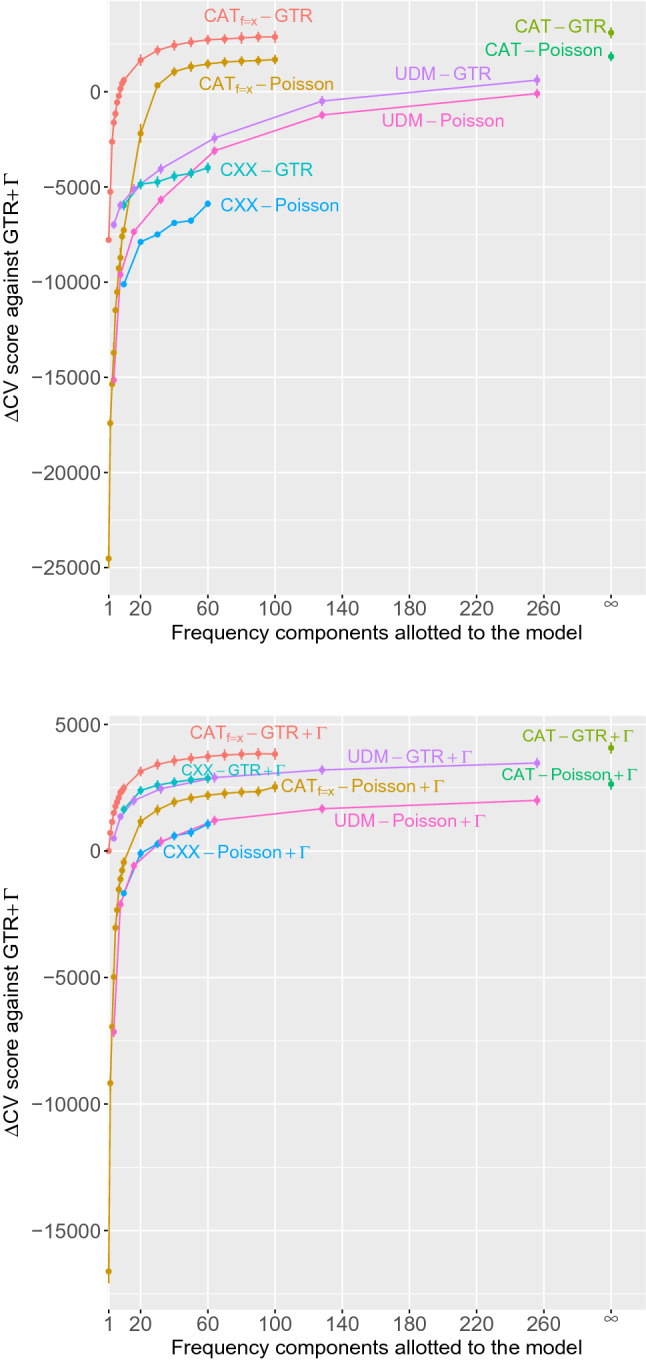
Cross-validation score (relative to the GTR$+\Gamma$ model) for the Lartillot-2007 data set, plotted as a function of the number of amino acid frequency components; the right-most abscissa marks show the results under CAT-based models. The top panel reports results for models without gamma-distributed rates-across-sites, whereas the bottom panel shows results for models with rates-across-sites. See materials and methods for descriptions of the models’ nomenclature

We used a fivefold cross-validation approach, randomly splitting each amino acid multiple sequence alignment into one fifth and four fifths (of the columns), using four fifths as the learning set, and the other fifth as the testing set. Note that the test dataset consists of columns that are not necessarily contiguous in the original alignment. We repeated such a random splitting five times. Overall, we thus performed five different training/testing runs for each data set. We computed the cross-validation score with the testing set as the log-summed site-specific likelihood averages over the sample from the posterior distribution on the learning set, as implemented in the PhyloBayes software (Lartillot et al. 2009; see material and methods section for details). The fivefold repetition provides a means of assessing the sampling variance in cross-validation scores associated with the random splitting of a data set. We repeated the learning and testing steps under each model included in our study. Finally, the entire procedure was repeated on five phylogenomic data sets referred to as Broughton, Brown, Delsuc, Lartillot-2007, and Lartillot-2012 (see materials and methods). A graphical representation of the results for one data set is shown in Fig. 1, and the detailed results across all data sets are given in Table 1. Within this table, we have displayed scores in bold when the model concerned is top-ranking in at least one of the five replicates of our overall cross-validation protocol.

### Empirical, Free Finite and Infinite Mixtures Comparisons

The infinite mixture models tend to outperform all finite mixture models. When comprised of a sufficiently high number of components—with a plateau typically reached between 80 and 100—the free finite mixture models CAT$_{f}$-Poisson and CAT$_{f}$-GTR approach closely their infinite mixture counterparts (Fig. 1, Table 1). However, it is difficult to anticipate the number of components required of a finite mixture model to approach the performance of the infinite mixture approach. The automatic shrinking effect of the infinite mixture model—naturally adapting to the level of heterogeneity under the given dataset—makes it a practical alternative to repeated applications of finite mixtures over a range of component numbers.

While the fluctuations in cross-validation scores across the five replicates do not always allow for a clear distinction between the top-performing models (Table 1), a look at the replicate-by-replicate performance of the models shows that CAT-GTR$+\Gamma$ is most often the best-performing choice. As reported in Table 2, for the smallest of the dataset (Broughton), two out five replicates have CAT-GTR$+\Gamma$ as the top model, two have CAT$_{f=100}$-GTR$+\Gamma$ (a free finite mixture with 100 components), and one has CAT$_{f=40}$-GTR$+\Gamma$ (a free finite mixture with 40 components). The other datasets all have CAT-GTR$+\Gamma$ performing best for the most replicates, with three of the dataset having five-out-of-five.

This strong performance of the CAT-GTR$+\Gamma$ model is in spite of an inherent potential disadvantage under the fivefold cross-validation method utilized here: richer models naturally require more data in order to provide reliable inferences, but since we are measuring the predictive power for a data set based on a learning step utilizing only four fifths of that data set, we risk underestimating the performance of infinite mixture models. In other words, the true overall performance of CAT-GTR$+\Gamma$ may be greater than the measurements we make here.

At an equal number of components, free finite mixture models always outperform the empirical models. The C10 to C60 empirical mixtures (Quang et al. 2008) never approach the cross-validation scores of the best-performing models (Table 1 reports the results of C60, the best-performing of these empirical mixture models, while Fig.1 shows a typical progression in cross-validation scores from C10 to C60). The UDM empirical mixtures (Schrempf et al. 2020), with many components, perform reasonably well, and in some cases, come close to matching the best free mixture models, albeit, with many more components (Table 1 reports the results of UDM256, the best performing in this class, while Fig.1 shows the progression in cross-validation scores across the range of UDM-models).

Overall, these results suggest that across-site heterogeneity in amino acid profiles is a highly pronounced feature of the amino acid replacement process, one that is well-expected from the variation in amino acid requirements across the sites of a protein. The results also suggest that there is still something elusive within the general project of constructing a “universal” profile mixture, since they are always outperformed by free mixtures.

### Mixture Models Versus Single-Matrix Models

As previously observed in several studies (e.g., Lartillot and Philippe 2004, 2006; Lartillot et al. 2007; Quang et al. 2008), the use of any mixture of amino acid profiles always leads to an improved predictive power of the model relative to the single-matrix counterpart (Table 1). This is obvious from the increasing cv-score as the number of mixture components grows from 1 to higher values, regardless of the type of finite mixture used (Fig. 1).

Interestingly, accounting for across-site pattern heterogeneity sometimes yields a greater improvement than accounting for rate heterogeneity. Specifically, for three data sets, a free mixture model (finite with a high number of components or infinite) on its own (with flat amino acid exchangeabilities and without gamma-distributed-rates) already outperforms the GTR$+\Gamma$ model; in the other two data sets, a free mixture model on its own matches closely the GTR$+\Gamma$ model (Table 1). A comparison between F81$+\Gamma$ versus CAT-Poisson shows that the latter provides a greater performance, altogether suggesting that modeling pattern heterogeneity has a greater impact on predictive power than modeling overall rate heterogeneity. Given the well-known importance of the gamma-distributed-rates model, and its nearly universal application in modern analyses, these results suggest that amino acid mixture models should perhaps also be considered a default choice.

### Empirical Mixture Models, Gamma-Distributed Rates, and Free Exchangeability Parameters

It is striking to note the extent to which the performance of empirical mixture models depends on coupling the approach to the gamma-distributed-rates and free exchangeabilities (Fig.1, Table 1). For two data sets (Broughton and Lartillot 2012), the UDM mixture model only clearly surpasses the single-matrix GTR$+\Gamma$ model when it is combined with both gamma-distributed rates and free amino acid exchangeabilities (Table 1). Across all data sets, the differences in cross-validation scores between empirical mixtures and free mixtures is greatly reduced by invoking the gamma-distributed-rates model. For instance, on the Lartillot-2007 data set, the difference in cross-validation score between CAT$_{60}$-GTR and UDM$_{64}$-GTR is around 5000 natural log units in favor of CAT$_{60}$-GTR, whereas the difference in score between CAT$_{60}$-GTR+$\Gamma$ and UDM$_{64}$-GTR+$\Gamma$ is only around 1000 units in favor of CAT$_{60}$-GTR+$\Gamma$ (Fig.1).

Coupling free amino acid exchangeability parameters with empirical mixtures provides an important means of modifying the effect of different mixture components in reaction to the data set under analysis. We speculate that if an empirical mixture does not include an appropriate component for a sufficiently large proportion of sites of the alignment, the model could react by adjusting the exchangeabilities between certain pairs of amino acids in ways that essentially break up few components into several; by having rates between key pairs very low, they become virtually inaccessible one from the other. Though it would still technically be an ergodic process, the ergodicity is, loosely speaking, impeded in the short term by the particular parameter configuration. Likewise, the gamma-distributed rates model could compensate for insufficiently specialized components by skewing the distribution of rates-across-sites one way or the other. With free mixture models, on the other hand, components adjust to the amino acid requirements of sites, sometimes in ways that implicitly capture overall rate heterogeneity; one way of having some sites with very low rates, for instance, is to have components dominated by a single amino acid. Indeed, for the Lartillot-2012 dataset, models without gamma-distributed-rates had the highest cross-validation score for two replicates (Table 2). Altogether, these different features could explain why empirical mixtures are so dependent on a coupling with other modeling approaches in order to achieve good performance, and free mixtures are not.

## Future Directions

Our results indicate that from the set of different modeling strategies, the use of mixtures of amino acid profiles to account for pattern heterogeneity has the highest contribution to the predictive power of a model of amino acid replacement. Moreover, while finite mixture models, including recent empirical mixtures, achieve strong performance, they still tend to be surpassed by the amino acid-level infinite mixture modeling using the Dirichlet process (Lartillot and Philippe 2004).

Wang et al. (2008) have reasonably suggested that mixture models might require fewer components when combined with free exchangeability parameters. Our results suggest that this is not necessarily the case. In fact, the improved performance across-the-board when invoking free exchangeability parameters suggests that even richer models along this direction could be warranted, perhaps including an independent mixture of exchangeability parameters.

The present study could also be extended to the comparison of other means of accounting for overall rate heterogeneity (e.g., Huelsenbeck and Suchard 2007), as well as partitioning approaches (Wang et al. 2019). Moreover, the set of models included here is limited to those assuming time-homogeneity, and future work should consider ways of comparing other models relaxing such assumptions (e.g., Blanquart and Lartillot 2008). A larger computational project could be undertaken to extend our study beyond five phylogenomic data sets to thousands in order to establish if our results are generalizable.

The focus of our study has been on the predictive power of models of the amino acid replacement process. Much more work remains, however, in order to assess how the suite of available models behave relative to phylogenetic inference *per se*. While single-gene-based studies, comparing different single-matrix models, suggest that model-fit does not reflect accuracy of phylogenetic inference (e.g., Spielman 2020), multi-gene applications of the mixture models considered here have known cases where higher model-fit translates to greater robustness against reconstruction artifacts, sometimes with profound biological implications (e.g., Lartillot et al. 2007; Feuda et al. 2017; Redmond and McLysaght 2021). One way of further characterizing such differences in phylogenetic inferences could be inspired by our approach of progressively scanning the finite mixture model space, to the point where finite mixtures converge to the CAT model, locating along the way the tipping-points of topological inferences with respect to mixture richness.

Finally, methodological work exploring different protocols of Bayesian cross-validation, and other means of model comparison, would be pertinent in better understanding their conclusions in practice. Exploring means of absolute model performance like posterior predictive checking, rather than constructing a simple ranking, will also be important in order to uncover which aspects of the amino acid replacement process require more attentive modeling.

## Materials and Methods

### Data

We studied five previously published datasets, which we refer to using the last name of the first author (adding the year of publication to disambiguate).

- Broughton: A concatenation of 20 amino acid alignments (6060 sites in total) from 61 species of fish, taken from Broughton et al. (2013).
- Brown: A concatenation of 159 amino acid alignments (43,649 sites in total) from 23 taxa from Amoebozoa and Opisthokonta, from Brown et al. (2013).
- Delsuc: A concatenation of 146 amino acid alignments (33,800 sites in total) from 38 taxa from deuterostomes, protostomes and fungi, obtained from Delsuc et al. (2006).
- Lartillot-2007: A concatenation of 146 amino acid alignments (35,371 sites in total) from 37 taxa across Bilateria and fungi, studied in Lartillot et al. (2007).
- Lartillot-2012: A concatenation of 17 amino acid alignments (5,039 sites in total) from 78 placental mammals, taken from Lartillot and Delsuc (2012).

### Models

The richest model we invoke, CAT-GTR$+\Gamma$, has been described in detail (Lartillot and Philippe 2004, 2006). Briefly, it consists of the GTR$+\Gamma$ model but with multiple sets of amino acid frequency parameters, following a Dirichlet process. The CAT-Poisson$+\Gamma$ model is only different from CAT-GTR$+\Gamma$ in having equal amino acid exchangeability parameters.

Free finite mixtures of similar form are written as CAT$_f$-GTR$+\Gamma$ in general, or as, say, CAT$_{f=100}$-GTR$+\Gamma$ for a free finite mixture with 100 components. As before, we write CAT$_{f}$-Poisson$+\Gamma$ when simplifying the model to even exchangeabilities.

Our notation of empirical mixture models replaces CAT with, say, C60, for the empirical mixture with 60 components proposed by Quang et al. (2008), with other elements of the notation as before (we say CXX to refer to this class of model in general). Likewise, we replace CAT with UDM256 for the empirical mixture proposed by Li et al. (2021) with 256 components. Note that in spite of using the empirical profile mixture values as provided by the respective authors, the weights of the mixture are treated as free parameters.

We write F81$+\Gamma$ for a very simple model based on a single set of amino acid frequency parameters with even exchangeabilities (analogous to the nucleotide-level model proposed by Felsenstein 1981). Finally, we omit the $+\Gamma$ to indicate that the model assumes homogeneous overall rates-across-sites.

### Bayesian Cross-Validation

We used a fivefold cross-validation approach. We first randomly select one fifth of the (not necessarily contiguous) amino acid columns in the multiple sequence alignment, which we set aside as the *testing* data set. The remaining four fifths of the amino acid columns then constitute the *learning* data set. We ran PhyloBayes on the learning data set under each model, with MCMC sampling for 2200 cycles on the *learning* data, and discarded 200 cycles as burn-in. We repeated this random data sub-sampling, and posterior sampling conditional on the learning data set, five times.

On the post-burn-in cycles, we used PhyloBayes to compute site-specific likelihood values over the sample on the testing data set, taking the averages for each site, and finally summing the logarithm of these site-specific likelihood posterior averages to produce the cross-validation score of each replicate. Supposing a sample of *K* (post-burn-in) parameter values drawn from the posterior distribution under the learning dataset, we denote a particular draw as $\theta _k$, where $1\le k \le K$. Writing $D_i$ for the *i*th column of the test data set, the likelihood score at site *i* given $\theta _k$ is written as $p(D_i \mid \theta _k)$. The Monte Carlo approximation of the Bayesian cross-validation score is given as:

$$\begin{aligned} \text {cv-score} = \sum _i \ln \left( \frac{1}{K} \sum _{k=1}^K p(D_i \mid \theta _k)\right). \end{aligned}$$

We compute the difference between the cross-validation score of a model of interest and the GTR$+\Gamma$ model, used as a reference, and report the average and standard deviation across the five replicates of the fivefold protocol. We also track the models receiving the highest cross-validation score for each of the five replicates.

Our choice of fivefold cross-validation, rather than two-fold, ten-fold, or other, is arbitrary, but attempts to balance a trade-off. On one hand, having a large test set means that the richer models are placed at a more significant disadvantage, since this implies a small learning set: richer models tend to require more training data to reliably infer parameter values. One the other hand, a very small test set will likely lead to a high variance in cross-validation scores across replicates, potentially making it difficult to clearly distinguish between all models’ performances. Results suggests that we can reasonably distinguish between most models with the fivefold protocol adopted, but more work is warranted to explore other protocols of data partitioning into learning and test sets.

**Table 1:** Cross-validation scores

	Broughton	Brown	Delsuc	Lartillot-2007	Lartillot-2012
F81	− 3641.6 ± 60.4	− 22518.4 ± 96.3	− 20009.6 ± 342.4	− 24521.0 ± 482.9	− 5579.5 ± 95.9
C60-Poisson	− 1520.8 ± 83.2	4341.4 ± 44.8	− 5084.2 ± 128.2	− 5881.2 ± 139.5	− 2733.2 ± 120.4
C60-GTR	− 912.4 ± 77.2	− 2003.2 ± 207.9	− 3454.0 ± 139.0	− 3996.6 ± 240.3	− 871.5 ± 77.3
UDM$_{256}$-Poisson	− 616.7± 69.1	1227.2 ± 52.2	36.4 ± 91.0	− 95.6 ± 206.4	− 1524.5 ± 87.3
UDM$_{256}$-GTR	− 238.9 ± 64.7	1746.6 ± 73.4	589.2 ± 121.2	610.6 ± 249.1	− 75.0 ± 45.0
CAT$_{f=100}$Poisson	− 66.7 ± 39.6	2859.8 ± 37.8	2028.2 ± 137.1	1692.6 ± 214.0	− 69.3 ± 65.4
CAT$_{f=90}$GTR	216.5 ± 47.7	3650.0 ± 176.8	2684.4 ± 126.0	2878.2 ± 229.5	**560.4** ± **31.1**
CAT$_{f=100}$GTR	262.4 ± 32.7	3617.4 ± 205.8	2716.8 ± 127.5	2878.0 ± 274.7	607.0 ± 18.1
CAT-Poisson	− 78.3 ± 37.6	2988.8 ± 44.8	2228.2 ± 147.0	1852.8 ± 211.1	− 65.2 ± 70.1
CAT-GTR	251.9 ± 34.9	3315.6 ± 63.8	2961.0 ± 151.6	3096.8 ± 249.7	**610.6** ± **15.8**
F81+$\Gamma$	− 2194.9 ± 52.5	− 15321.4 ± 215.7	− 13044.0 ± 213.4	− 16612.6 ± 441.8	− 3607.9 ± 65.3
C60-Poisson+$\Gamma$	− 170.9 ± 44.7	2227.6 ± 48.5	1435.8 ± 107.5	1061.2 ± 165.0	− 1038.1 ± 84.0
C60-GTR+$\Gamma$	253.6 ± 16.6	3193.2 ± 34.0	2686.4 ± 105.2	2869.6 ± 162.8	370.5 ± 34.8
UDM$_{256}$-Poisson+$\Gamma$	− 39.9 ± 35.2	2962.8 ± 46.9	2306.0 ± 123.6	1996.4 ± 166.5	− 868.8 ± 83.7
UDM$_{256}$-GTR+$\Gamma$	323.4 ± 20.6	3651.2 ± 41.6	3204.0 ± 139.8	3473.6 ± 169.9	427.0 ± 41.7
CAT$_{f=100}$Poisson+$\Gamma$	65.0 ± 40.1	3294.2 ± 62.7	2627.2 ± 158.6	2529.6 ± 186.3	13.7 ± 58.4
CAT$_{f=40}$GTR+$\Gamma$	**353.1** ± **23.6**	3599.4 ± 27.2	3237.0 ± 102.6	3563.8 ± 179.7	590.0 ± 36.4
CAT$_{f=100}$GTR+$\Gamma$	**374.9** ± **20.8**	3789.2 ± 43.1	3446.8 ± 109.7	3824.8 ± 203.7	**624.0** ± **31.9**
CAT-Poisson+$\Gamma$	57.5 ± 40.6	3404.8 ± 56.1	2820.0 ± 150.5	2638.8 ± 188.1	30.5 ± 66.5
CAT-GTR+$\Gamma$	**370.9** ± **24.2**	**3943.2** ± **36.8**	**3678.6** ± **134.5**	**4069.8** ± **196.2**	**619.7** ± **36.6**

Models with an instance of the highest performance in at least one replicate are displayed in bold. For empirical mixtures, only results for the top-performing model are displayed. For free finite mixture models, results for 100 components are displayed, as well as any free finite mixture having the best performance on at least one of five replicates

**Table 2:** Number of replicates where a model had the best performance

	Broughton	Brown	Delsuc	Lartillot-2007	Lartillot-2012
CAT-GTR+$\Gamma$	2	5	5	5	2
CAT-GTR					1
CAT$_{f=100}$GTR+$\Gamma$	2				1
CAT$_{f=40}$GTR+$\Gamma$	1				
CAT$_{f=90}$GTR					1
